# Usefulness of Portable Device to Establish Differences in Muscle Oxygenation Between the Wingate Test and Graded Exercise Test: Effect of Gender on Anaerobic and Aerobic Capacity in Speed Skaters

**DOI:** 10.3389/fphys.2022.809864

**Published:** 2022-03-08

**Authors:** Kinga Rębiś, Dorota Sadowska, Michal Starczewski, Andrzej Klusiewicz

**Affiliations:** ^1^Department of Physiology, Institute of Sport - National Research Institute, Warsaw, Poland; ^2^Faculty of Rehabilitation, Józef Piłsudski University of Physical Education in Warsaw, Warsaw, Poland; ^3^Faculty of Physical Education and Health, Biała Podlaska, Józef Piłsudski University of Physical Education in Warsaw, Warsaw, Poland

**Keywords:** speed skaters, supramaximal and maximal exercise tests, muscle oxygenation, anaerobic and aerobic capacity, portable oxygenation devices

## Abstract

The aim of this study was to compare the oxygenation response in the vastus lateralis muscle (SmO_2_) in two types of tests: supramaximal and maximal. Furthermore, gender differences in SmO_2_ response to test exercise were assessed and the usefulness of muscle oxygenation measurements in the assessment of anaerobic and aerobic capacity was determined. The Wingate test (WAnT) and the graded exercise test (GXT) to exhaustion were performed on a cycle ergometer to examine 13 female and 14 male speed skaters from the junior and U23 national teams. During both tests, SmO_2_ of the vastus lateralis muscle was recorded by near-infrared spectroscopy at baseline (at rest or post warm-up), at exercise, and during recovery. The most significant SmO_2_ indices (differences between baseline and post-exercise indices and half time for SmO_2_ to reach the maximal post-exercise value) were not significantly different between the tests. Gender was also not a differentiating factor in muscle oxygenation regardless of test type. In the GXT test, half time required for SmO_2_ to reach the maximal value correlated negatively with VO_2max_ and test duration, thus confirming the usefulness of SmO_2_ measurements in the assessment of the aerobic capacity of speed skaters. In contrast, the WAnT test showed no significant correlations between exercise indices and muscle oxygenation indices. From the standpoint of the assessment of anaerobic capacity, SmO_2_ measurements showed little diagnostic value.

## Introduction

Periodic physical capacity testing of speed skaters often includes maximal aerobic testing with a graded load (Stangier et al., 2016) and supramaximal tests assessing anaerobic exercise capacity (Hofman et al., 2017). The aerobic contribution increases with the length of the race distance, but no relationship has been demonstrated between the level of the maximal oxygen uptake and the athletic performance of speed skaters (Konings et al., 2015). Furthermore, speed skaters generate high power output during races, which requires adequate levels of aerobic and anaerobic capacity, although their proportional contribution varies depending on the race distance (Konings et al., 2015; Piucco et al., 2018). On the other hand, an assessment of short-term exercise capacity is particularly relevant in speed skating for athletes competing over short distances, who generate the highest power values (Konings et al., 2015). According to the literature, skaters who score high on the Wingate test are also more successful during competitions (Nagasawa, 2013).

The latest near-infrared spectroscopy (NIRS) devices measuring muscle oxygenation (SmO_2_) are useful in the study of speed skaters, especially when conducted on ice (Hesford et al., 2012; Hettinga et al., 2016). The specific position that is necessary for speed skating imposes certain physiological limitations. Prolonged isometric muscle contractions and a sitting low position impede blood flow to the lower limb muscles, reduce quadriceps oxygenation, and increase blood lactate levels. This impairs oxygen transport and leads to increasing fatigue processes (Hesford et al., 2013). Despite previously conducted research on ice, there remains several essential queries related to laboratory tests and NIRS measurements. In the present study, we sought to investigate whether changes in locomotory muscle oxygenation levels are dependent on the type of test conducted under laboratory conditions when assessed with NIRS. Understanding, how supramaximal and maximal exercise intensities impact muscle oxygenation in speed skating can help to deliver training monitoring methods based on SmO_2_ measurements.

It should be emphasized that NIRS measurements are also increasingly used during testing in other sports such as cycling, cross-country running, and kayaking (Borges and Driller, 2016; Crum et al., 2017; Paquette et al., 2018). In the last few years, the introduction of mobile oximeters to measure the oxygenation level of myoglobin in the muscle cytoplasm and hemoglobin in the blood vessels of the muscle microcirculation (muscle oxygen saturation, SmO_2_) has been particularly relevant to training practice (Perrey and Ferrari, 2018). SmO_2_ measurements during exercise provide valuable information about oxygen transport and utilization in muscles (McCully and Hamaoka, 2000). Recovery time of muscle reoxygenation after submaximal or maximal exercise has been associated with muscle oxidative capacity. An increase in recovery time indicates an oxygen deficit, especially when exercise intensity increases (Hamaoka, 2013). In speed skating, the most common location for the NIRS monitor is vastus lateralis (Hesford et al., 2012; Hettinga et al., 2016). These measurements do not require blood sampling and are not associated with high financial costs, which is why, in recent years, there has been an increasing interest in this subject from both researchers and coaches. According to many authors, NIRS measurements are a simple, safe, and fast way to assess exercise capacity during short-term maximal contractions (Cettolo et al., 2007) and to determine so-called training intensity zones (Borges and Driller, 2016; Driller et al., 2016; Crum et al., 2017). For the training process, it is important to know not only the global maximal parameters of the athlete, such as work rate, power, or maximal oxygen uptake, but also to understand the body’s response to the effort at the local level, as assessed by NIRS. There have already been attempts to study muscle oxygenation in speed skaters (Hesford et al., 2012, 2013; Hettinga et al., 2016; Piucco et al., 2018) indicating the importance of studies assessing the relationship between changes in SmO_2_ and locomotor muscle fatigue in this sport. Despite the successful use of NIRS in studies on speed skaters, the differences in the SmO2 changes between an aerobic and an anaerobic test has not been characterized in a group of high-performance athletes. It is therefore justified to continue the analysis using NIRS in high-performance speed skaters after various types of exercise, which has both diagnostic and practical significance in the selection of training loads (Hettinga et al., 2016).

An important aspect of our research was therefore the combination of classical physical laboratory tests performed periodically by speed skaters with measurements of muscle oxygenation. The main aim of this study was therefore to compare the oxygenation response of the vastus lateralis muscle in two types of tests: supramaximal and maximal, to determine the usefulness of NIRS measurements in assessing anaerobic and aerobic capacity in speed skaters. Furthermore, gender differences in SmO_2_ response to test effort in athletes were also evaluated, which, to our knowledge, has not yet been described in the literature. We have found only one recent published research that attempts to assess muscle oxygenation responses in relation to gender, but unlike the current study, it involved physically active participants, not athletes (Espinosa-Ramírez et al., 2021).

## Materials and Methods

### Subjects

The study involved 27 athletes (13 females and 14 males), who were the leading long track Polish speed skaters of junior and U23 national teams, aged 14.8–22.1 years. Training experience was 4.2 ± 1.5 in the group of female skaters and 3.5 ± .9 years in male skaters. Both female and male skaters practiced different speed skating events: sprint (11 skaters), middle-distance (9), and long-distance (7) speed skating events. Characterization of the study participants is presented in Table 1. Athletes performed two exercise tests on 1 day, with a minimum 2-h break between tests. Measurements of muscle oxygenation and classical exercise indices determined by standard exercise tests (the Wingate test and graded exercise test) used in speed skaters were analyzed.

**Table 1:** Basic characteristics of the examined speed skaters (*n* = 27), mean ± SD.

Variable/group	Female (*n* = 13)	Male (*n* = 14)
Age (years)	18.5 ± 2.0	17.9 ± 2.2
Body height (cm)	168.2 ± 3.	182.0 ± 6.8
Body weight (kg)	60.4 ± 3.6	73.9 ± 6.5
BMI (kg/m^2^)	21.3 ± 1.0	22.3 ± 1.2
Training experience (years)	4.2 ± 1.5	3.5 ± .9

The study was conducted in cooperation with National Speed Skating Association during the preparatory training period (in September) of the annual training plan. The presented study was conducted according to the guidelines of the Declaration of Helsinki (1975) and was approved by the Institutional Research Ethics Committee.

Athletes were selected for the study by national teams coaches and participated voluntarily. They were informed about the right to withdraw at every stage of the research. All participants provided written consent for participation, and in the case of underage, such consent was received from their legal guardians.

### Exercise Tests

The anaerobic capacity test (the Wingate Test, WAnT) was performed first on a Monark 874E cycle ergometer (Monark Exercise AB, Sweden). This test was preceded by a standard 5-min warm-up with a load of 1.0 W/kg. Next, after 2 min of rest, the athlete performed a 30-s test ith the load individually adjusted to body weight (7.5% of body weight). The subject’s task was to produce the highest possible cadence after the “Start!” command and to maintain it as high as possible for the test duration. The speed skaters were instructed not to raise their hips from the saddle but to always make their best effort to pedal during the Wingate test. The basic mechanical parameters of the effort, i.e., work done, maximum power, and fatigue index, were computed using a computer program coupled with the cycle ergometer (MCE 5.1—JBA Z. Staniak, Poland).

The maximal graded exercise test (GXT) was performed on a Lode Excalibur Sport PFM cycle ergometer (Lode B. V., Netherlands). The test consisted of 3-min stages with graded load performed continuously. The test was started in male and female skaters with a load of 1.0 W/kg and the load was increased every stage by .7 W/kg (female skaters) or .8 W/kg (male skaters). During the test, heart rate (HR) was continuously recorded using the H9 Heart Rate Sensor (Polar Electro Oy, Finland). Respiratory gases were measured during each testing session using wearable and wireless breath-by-breath pulmonary gas analyzer (Metamax 3B, Cortex Biophysik GmbH, Germany), previously demonstrated to be reliable for measuring oxygen uptake (Overstreet et al., 2017).

After completing both tests, the athletes continued pedaling for active cool-down (light exercise at a load of .5 W/kg performed for 3 min after the completion of the test) aimed at avoiding an orthostatic shock.

### Maximal Aerobic Power

Maximal aerobic power (*P*_max_) was calculated as a proportion of the time and a power of the last executed bout in the GXT (Kuipers et al., 1985). The equation for calculation is presented below:


$$P_{max}=P_{LFE}+\left(T_{LE}/T_{St}*\Delta P\right).$$

*P*_max_—maximal aerobic power; *P*_LFE_—power of the last fully executed step; *T*_LE_—time executed in the final step; *T*_St_—time of the last step; and Δ*P*—increase in power between last two steps.

### Maximal Oxygen Consumption

Maximal oxygen uptake (VO_2max_) was defined as the highest amount of oxygen consumption over a 30-s period during the test. The maximal intensity exercise necessary for the estimation of VO_2_max was defined by the following criteria: the VO_2_ plateauing with increasing workload, the post-exercise blood lactate concentration > 8 mmol/l, the respiratory exchange ratio (RER) >1.1, and attainment of the age-adjusted maximal heart rate expressed as HRmax = 220—participant age. If at least two of the above criteria were met during the exercise, the attained effort and oxygen uptake were regarded as maximal.

### Measurements of Muscle Oxygenation

During both tests, a NIRS device (Moxy monitors; Fortiori Design LLC, Hutchinson, MN, United States) was placed on the vastus lateralis (VL) muscle that is active during cycling. The Moxy monitor is a continuous wave near-infrared spectroscopy monitor. It uses a new type of algorithm that is based on Monte Carlo modeling. The system uses four wavelengths at 680, 720, 760, and 800 nm. The spacings between two emiters and detector are 12.5 and 25 mm respectively. The device was approximately 15 ± 2 cm above the proximal border of the patella on the vastus lateralis muscle belly and was fixed to the right limb with a dark 7.5 cm dynamic tape by the same person. The muscle oxygen saturation (SmO_2_) was recorded at baseline (at rest for GXT or post warm-up for WAnT), during exercise, and during the recovery period. SmO_2_ reflects the dynamic balance between oxygen (O_2_) consumption and supply (Ichimura et al., 2006). SmO_2_ measurements taken 2 min after the warm-up and before the test were taken as baselinevalues for WAnT and GXT, respectively. The differences in SmO_2_ (Δ SmO_2_) between baseline and minimal exercise levels were also calculated. The reoxygenation rate after both tests were evaluated as the half time required for SmO_2_ recovery (SmO_2_ HTR; McCully et al., 1994; Nagasawa, 2013). The SmO_2_ data values for further calculations were averaged from measurements made 2.0 min before tests (SmO_2_ baseline), during 2 s at exercise minimum (SmO_2_ minimum), and recovery maximum (SmO_2_ maximum) The mean SmO_2_ for 2 s immediately after the completion of the exercise was defined as 0% and the maximum SmO_2_ in the first 3 min of the recovery phase after completion of exercise was defined as 100%. The SmO_2_ HTR was then defined as the time from the completion of exercise to the time to reach 50% SmO_2_.

### Statistical Analysis

The statistical analyses were conducted with the Statistica 13.0 software. The normal distribution of variables was examined using the Shapiro–Wilk test. The significance of differences was assessed using the Student test and Mann-Whitney U-test for variables with a distribution different from the normal distribution. The power of relationships between the variables was determined based on Spearman’s rank correlation coefficients (*R*). The coefficient intervals for correlations were also presented. The level of statistical significance was set at *p* ≤ .05.

## Results

No significant differences in muscle oxygenation of the vastus lateralis muscle indices were observed between female and male speed skaters during both tests (see Tables 2 and 3).

**Table 2:** Values of selected indices recorded during the Wingate test in the examined group of female and male speed skaters (*n* = 27), mean ± SD.

Variable/group	Female (*n* = 13)	Male (*n* = 14)	*p*
Power max (W)	676 ± 55	916 ± 100	≤.0001
Power max (W/kg)	11.2 ± .7	12.4 ± .8	.0004
Work (J)	16,130 ± 925	21,768 ± 2,300	.0003^U^
Work (J/kg)	268 ± 15	297 ± 15	≤.0001
SmO_2_ baseline (%)	59.3 ± 6.5	61.2 ± 6.2	.4501
SmO_2_ minimum (%)	8.6 ± 3.5	9.7 ± 5.2	.5478
SmO_2_ maximum (%)	70.3 ± 9.6	71.6 ± 4.5	.6605
∆ SmO_2_ (%)	50.7 ± 8.1	51.5 ± 7.9	.7853
SmO_2_ HTR (s)	24.3 ± 11.4	22.7 ± 4.5	.6799^U^

*∆ SmO_2_ (%)—SmO_2_ difference between baseline and minimum values. SmO_2_ HTR—half time for SmO_2_ to reach the maximal value. p—difference between female and male skaters*.

U *Mann–Whitney U test.*

**Table 3:** Values of selected indices recorded during the graded exercise test in the examined group of female and male speed skaters (*n* = 27), mean ± SD.

Variable/group	Female (*n* = 13)	Male (*n* = 14)	*p*
P_max_ (W)	243 ± 18	353 ± 45	≤.0001
P_max_ (W/kg)	4.04 ± .33	4.77 ± .43	≤.0001
VO_2_max (l/min)	3.18 ± .26	4.53 ± .42	≤.0001
VO_2_max (ml/kg/min)	52.1 ± 4.6	62.0 ± 4.8	≤.0001
SmO_2_ baseline (%)	65.2 ± 9.6	64.3 ± 7.7	.7825
SmO_2_ Minimum (%)	13.4 ± 6.8	12.3 ± 6.6	.6917
SmO_2_ Maximum (%)	76.5 ± 5.2	76.6 ± 5.7	.9316
∆ SmO_2_ (%)	51.9 ± 10.7	52.0 ± 11.0	.5934^U^
SmO_2_ HTR (s)	29.8 ± 13.1	22.6 ± 8.7	.0894^U^

*P_max_—maximal aerobic power. ∆ SmO_2_ (%)—SmO_2_ difference between baseline and minimum values. SmO_2_ HTR (s)—half time for SmO_2_ to reach the maximal value. p—difference between female and male skaters*.

U *Mann–Whitney U test.*

**Figure 1:**
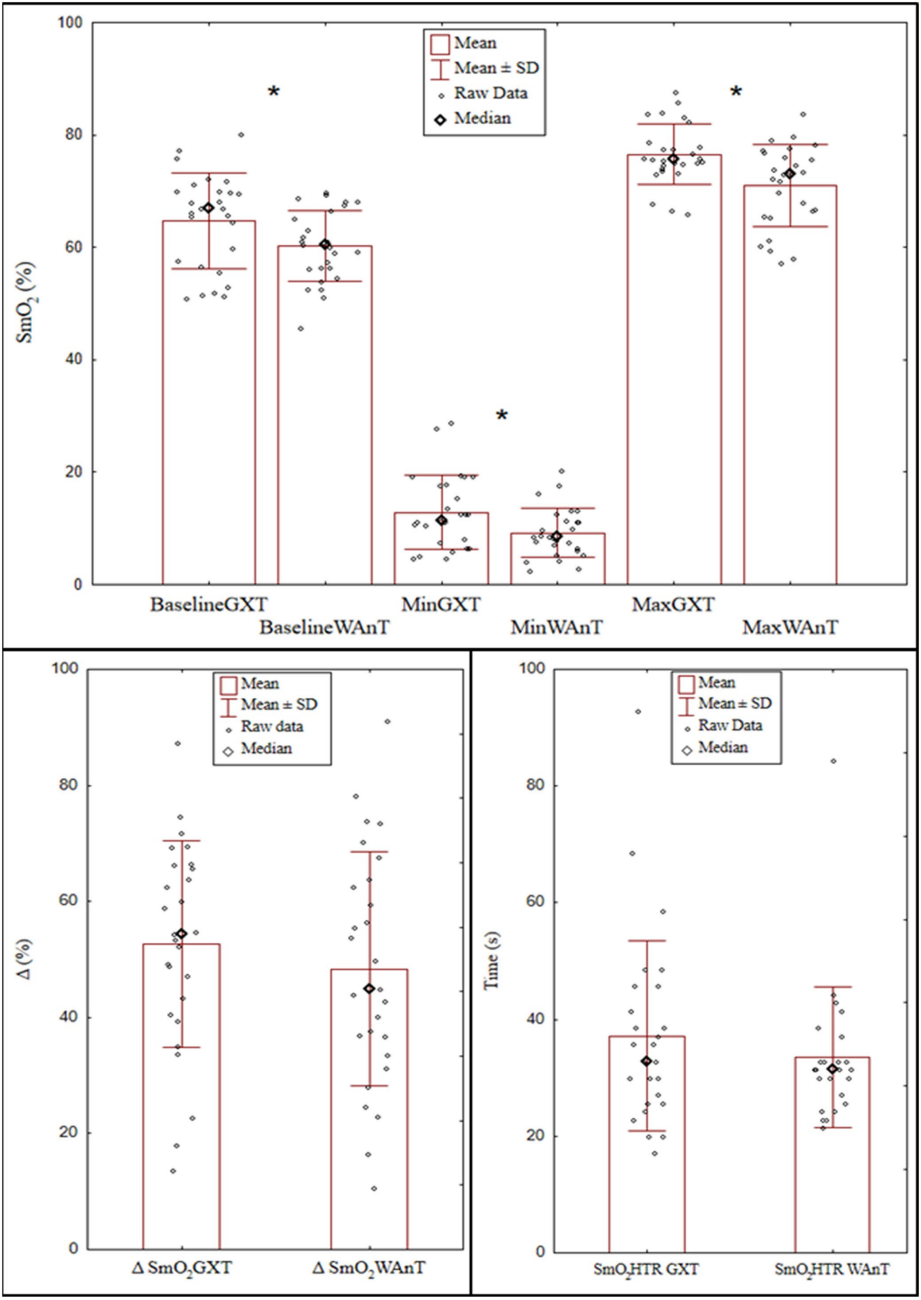
Muscle oxygen saturation (SmO_2_): at baseline, as exercise minimum values, post exercise maximum values, differences between baseline and minimum values (Δ SmO_2_) and half time for SmO_2_ to reach the maximal value (SmO_2_ HTR) during the graded exercise test (GXT) and the Wingate test (WAnT) in the combined group female and male speed skaters (*n* = 27). ^*^Mann-Whitney U test (*p* < .05).

During WAnT for the combined analysis of male and female results, a rapid real-time decline was found in SmO_2_, lasting until the end of the test. Average ∆SmO_2_ across the whole group (*n* = 27) was 51.1 ± 7.8% (Figure 1).

SmO_2_ HTR was 23.5 ± 8.5 s on average. During the GXT, ∆SmO_2_, and SmO_2_HTR were 51.9 ± 10.6% and 26.0 ± 11.4 s, respectively. While ΔSmO_2_ and SmO_2_HTR did not differ between the WAnT and the GXT, significant differences were found in baseline, minimum, and maximum SmO_2_ levels, which were significantly lower after WAnT (60.2 ± 6.3%,9.2 ± 4.4%, and 70.9 ± 7.3, respectively) compared to GXT (by 64.7 ± 8.5%, 12.8 ± 6.6%, and 76.5 ± 7.3, respectively; Figure 1).

During WAnT, no significant correlations were observed between muscle oxygenation indices and exercise capacity indices (Power Max, Work, and Fatigue Index), (Table 4).

**Table 4:** Spearman’s rank correlation coefficients values between selected indices recorded during the Wingate test in the examined group of female and male speed skaters (*n* = 27).

Variable	Power Max		Work		Fatigue Index	
	(W)	(W/kg)	(J)	(J/kg)	%	W/s
∆ SmO_2_ (%)	.05 (−.34–.42)	.34 (−.05–.64)	.10 (−.29–.46)	.31 (−.08–.62)	−.36 (−.65–.02)	−.19 (−.53–.20)
SmO_2_ HTR	.10 (−.29–.46)	.05 (−.34–.42)	.06 (−.33–.43)	.02 (−.36–.40)	.20 (−.19–.54)	.02 (−.36–.40)
SmO_2_ minimum (%)	.13 (−.26–.49)	.03 (.35–.41)	.13 (−.26–.49)	.02 (−.36–.40)	.17 (−.22–.52)	.16 (−.23–.51)

*The numbers in the table presenting r values and coefficient interval for r in the brackets. ∆ SmO_2_ (%)—SmO_2_ difference between resting and minimum values. SmO_2_ HTR—half time for SmO_2_ to reach the maximal values.*

Furthermore, in this test, muscle oxygenation indices did not correlate significantly with VO_2_max expressed in absolute values and calculated per body weight (Table 5).

**Table 5:** Spearman’s rank correlation coefficient values between VO_2_maxand Pmax and selected indices of muscle oxygenation recorded during the Wingate and graded exercise tests in the group of female and male speed skaters (*n* = 27).

Variable	VO_2_max		Pmax	
	(l/min)	(ml/kg/min)	W	W/kg
**Wingate test**				
SmO_2_ minimum (%)	.05 (−.42–.34)	−.01 (−.39–.37)	.17 (−.22–.52)	.19 (−.20–.53)
∆ SmO_2_ (%)	.08 (−.31–.45)	.10 (−.29–.46)	−.11 (−.47–.28)	−.07 (−.44–.32)
SmO_2_ HTR	−.18 (−.21–.52)	−.17 (−.52–.22)	−.19 (−.53–.24)	−.33 (−.63–.06)
**Graded exercise test**				
SmO_2_ Minimum (%)	−.06 (−.43–.33)	−.28 (−.60–.11)	.15 (−.24–.50)	.06 (−.33–.24)
∆ SmO_2_ (%)	.10 (−.29–.46)	.19 (−.20–.53)	−.03 (−.41–.35)	−.04 (−.41–.34)
SmO_2_ HTR	−.40^*^ (−.68 to −.02)	−.39^*^ (−.67 to −.01)	−.40^*^ (−.68 to −.02)	−.49^*^ (−.73 to −.14)

*The numbers in the table presenting r values and coefficient interval for r in the brackets. ∆ SmO_2_ (%)—SmO_2_ difference between baseline and minimum values. SmO_2_ HTR—half time for SmO_2_ to reach the maximal values*.

* *p < .05*.

**Figure 2:**
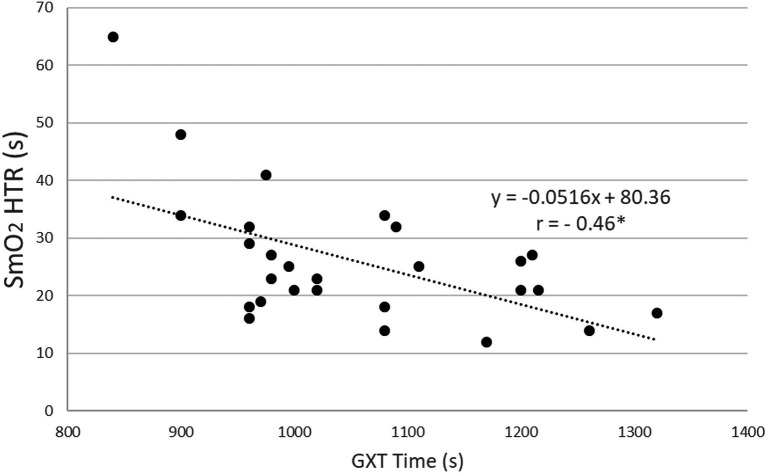
Statistically significant Spearman’s rank correlation between half time for SmO_2_ to reach the maximal value (SmO_2_ HTR) and test time in the graded exercise test (GXT) in the combined group of female and male speed skaters (*n* = 27). ^*^p < .05.

In GXT, significant correlations with muscle oxygenation indices were observed between SmO_2_HTR, maximal oxygen uptake, and maximal power in absolute (*r* = −.40, and *r* = −.40, respectively, *p* < .05) and relative (*r* = −.39, *r* = −.49, respectively, *p* < .05) values (Table 5), and test duration (*r* = −.46, *p* < .05), (Figure 2).

## Discussion

An important observation was that there were no significant gender differences in the muscle oxygenation response to anaerobic and aerobic exercise. This is related to the fact that regardless of gender, factors that improve oxygen delivery to working muscles, such as muscle capillary density, blood volume, plasma volume influencing cardiac output, red blood cell volume, or total hemoglobin mass, improve with increased fitness level (Heinicke et al., 2001; Malczewska-Lenczowska et al., 2013; Steiner et al., 2019; Zelenkova et al., 2019). Importantly, our study reports that the rate of SmO_2_ reoxygenation showed a significant correlation with VO_2_max and exercise capacity in GXT. In speed skaters with higher maximal oxygen uptake and test duration, the quadriceps muscle oxygenation returned to maximal levels faster after GXT. Similarly, Ichimura et al. (2006) also showed that the rate of muscle reoxygenation after a ramp test was faster in subjects with higher VO_2_max levels. It should be emphasized that the reoxygenation times obtained in both tests were affected by active cool-down. These results suggest that the rate of muscle reoxygenation in the graded exercise test depended on the aerobic capacity of speed skaters.

The participants in our study were selected Polish speed skaters of different ages and speed skating events (sprinters, middle-distance, and long-distance skaters). They were junior and U23 speed skaters with a maximal oxygen uptake of 52.1 ± 4.6 in female skaters and 62.0 ± 4.8 ml/kg/min in male skaters. A review of the literature suggests that skaters achieve relatively low values of maximal oxygen uptake in a test performed on a cycle ergometer compared to other endurance sports (between 52.2 and 54.9 ml/kg/min for females and 57.2 and 62.0 ml/kg/min for male skaters; Konings et al., 2015).

We observed that SmO_2_ indices show little diagnostic value for the assessment of anaerobic capacity in highly trained athletes, indicating a lack of significant correlation with both the maximal power and work in WAnT (Table 4). Another study (Nagasawa, 2013) indicated a slower recovery rate of muscle oxygenation after WAnT in long-distance runners compared with that in sprinters and healthy controls. In contrast to this study, a larger group of female and male speed skaters did not show a higher reoxygenation rate after an analogous exercise (WAnT) in participants with lower VO_2_max expressed both in absolute values and converted to body weight (Table 5). While the usefulness of SmO_2_ is reduced for the assessment of anaerobic capacity in speed skaters, this does not exclude that such measurements may be important in the choice of the training load, e.g., during high-intensity intervals. However, it should be mentioned that this problem was not the subject of our research. In presented research SmO_2_ indices, maximal power and work in WAnT, maximal power, and maximal oxygen uptake in GXT were determined. To our knowledge, this is the first research paper comparing SmO_2_ responses in speed skaters between two different types of exercise tests. Previous studies (Buchheit and Ufland, 2011; Nagasawa, 2013; Paquette et al., 2020; Vasquez-Bonilla et al., 2021) examined recovery following WAnT and short-term efforts in kayak and football players, or the rate of reoxygenation following maximal aerobic exercise test (Ichimura et al., 2006). One of the study’s limitations is the differences in baseline SmO_2_ values found among speed skaters could be due to the slightly different phase of measurement, i.e., 2 min after the warm-up in WAnT (affecting better blood supply and muscle oxygenation) and at rest before GXT, (Figure 1) or slight position change of the NIRS device. However, in our research, SmO_2_ post warm-up values in WAnT were lower than rest SmO_2_ before GXT, that is in contrast to previous observations. The short duration of the interval (2 min) between the warm-up and the WAnT test may have contributed to the lower than expected baseline SmO_2_ values. Additionally, it can be assumed that the described differences also contributed to the lower minimum SmO_2_ values after WAnT. Unlike previous studies by other authors (Nagasawa, 2013), the WAnT methodology intentionally used a warm-up as required by the methodology of performing this test. According to Frikha et al. (2016), the warm-up model used and the duration of the rest before performing 30 s of exercise has a significant effect on the results obtained and the assessment of anaerobic capacity.

The values of the remaining SmO_2_ indices, i.e., ΔSmO_2_ and SmO_2_HTR are not dependent on the type of test performed (Figure 1). One reason for the lack of differences may have been the rapid rate of decline in muscle oxygenation observed already several seconds after the start of exercise, which was important for the short-term WAnT test. Other cardiopulmonary indices such as heart rate and oxygen uptake require much longer exercise duration to reach their maximal values. These observations support the need for further research into local changes in oxygenation and their use in the control and monitoring of exercise load.

## Conclusion

This study shows that regardless of the type of test, the gender of speed skaters does not differentiate between muscle oxygenation responses. The rate of muscle reoxygenation in the graded exercise test depends on the aerobic capacity of speed skaters. However, SmO_2_ indices show less usefulness diagnostic value for assessing anaerobic capacity. Finally, the most significant SmO_2_ indices (differences between baseline and minimal SmO_2_ and half time for SmO_2_ to reach the maximal post-exercise levels) were not significantly different between the Wingate test and graded exercise test.

## Practical Applications

The use of non-invasive measurements of the rate of muscle reoxygenation can be useful in assessing the aerobic capacity of speed skaters, thus contributing to a more in-depth analysis of post-training adaptations.

## Limitation

The study group consisted of relatively few representatives of different speed skating events (sprint *n* = 11, middle distance *n* = 9, and long distance *n* = 7). In future research, it may be advisable to conduct similar analyses on larger groups of speed skaters with regard to individual events.

## Data Availability Statement

The raw data supporting the conclusions of this article will be made available by the authors, without undue reservation.

## Ethics Statement

The studies involving human participants were reviewed and approved by the Komisja Etyki Badań Naukowych, Instytut Sportu—Państwowy Instytut Badawczy (The Scientific Research Ethics Committee, Institute of Sport—National Research Institute). The patients/participants provided their written informed consent to participate in this study.

## Author Contributions

All authors listed have made a substantial, direct and intellectual contribution to the work, and approved it for publication.

## Funding

The study was conducted with financial support from the Ministry of Culture National Heritage and Sport of the Republic of Poland under grant number 2021/0408/Udot/32/DSW.

## Conflict of Interest

The authors declare that the research was conducted in the absence of any commercial or financial relationships that could be construed as a potential conflict of interest.

## Publisher’s Note

All claims expressed in this article are solely those of the authors and do not necessarily represent those of their affiliated organizations, or those of the publisher, the editors and the reviewers. Any product that may be evaluated in this article, or claim that may be made by its manufacturer, is not guaranteed or endorsed by the publisher.
